# Acceptability and Pilot Validation of the Diagnostic Autism Spectrum Interview (DASI-2) Compared with Clinical and ADOS-2 Outcomes

**DOI:** 10.3390/children12081025

**Published:** 2025-08-04

**Authors:** Susan Jane Young, Nóra Kollárovics, Bernadett Frida Farkas, Tímea Torzsa, Rebecca Cseh, Gyöngyvér Ferenczi-Dallos, Judit Balázs

**Affiliations:** 1 Independent Practice, Psychology Services Ltd., London, CR9 7AE, UK; 2 Department of Psychology, University of Reykjavik, Menntavegur 1, 110 Reykjavik, Iceland; 3 Doctoral School, Semmelweis University, 1085 Budapest, Hungary; kollarovics.nora@phd.semmelweis.hu (N.K.); farkas.bernadett@phd.semmelweis.hu (B.F.F.); 4 International Cseperedő Foundation, 1136 Budapest, Hungary; 5 Department of Psychiatry and Psychotherapy, Semmelweis University, 1082 Budapest, Hungary; 6 Doctoral School of Psychology, ELTE Eötvös Loránd University, 1064 Budapest, Hungary; torzsa.timea@ppk.elte.hu (T.T.); cseh.rebecca@ppk.elte.hu (R.C.); balazs.judit@ppk.elte.hu (J.B.); 7 Gildor Bt, Határőr út 55, 1122 Budapest, Hungary; dallos.gyongyver@ppk.elte.hu; 8 Department of Psychology, Oslo New University College, 0454 Oslo, Norway; 9 Lumos Child, Adolescent and Adult Psychiatry Outpatient Clinic, 1137 Budapest, Hungary

**Keywords:** autism, ASD, DASI, diagnosis, ADOS-2, clinical assessment

## Abstract

Background/Objectives: There is a growing need for autism spectrum disorder (ASD) assessment tools that are diagnostically aligned, clinically usable, and accessible across diverse service contexts. The Diagnostic Autism Spectrum Interview—Version 2 (DASI-2) is a freely available, semi-structured clinical interview mapped directly to DSM-5 and ICD-11 criteria. This pilot study aimed to adapt DASI-2 into Hungarian and explore the (1) acceptability of DASI-2 administration, (2) agreement with prior clinical ASD diagnoses, and (3) relationship between DASI-2 observational ratings and ADOS-2 classifications. Methods: Following a multistep translation procedure, DASI-2 was administered to seven children previously assessed for ASD in a multidisciplinary Hungarian clinical setting. The assessment included a parent interview, direct assessment with the child or young person, and completion of the DASI observational record (OR1–OR4). DASI diagnostic outcomes were compared with prior clinical decisions, and OR scores were analyzed in relation to ADOS-2 classifications. Results: All participants completed the DASI-2 interview in full. Agreement with prior clinical diagnosis was found in six of seven cases (κ = 0.70, indicating substantial agreement). When exploring the one non-aligned case, the divergence in diagnostic outcome was due to broader contextual information considered by the initial clinical team which influenced clinical opinion. The five participants diagnosed with ASD showed substantially higher DASI observational scores (mean = 15.26) than the two who were not diagnosed (mean = 1.57), mirroring ADOS-2 severity classifications. Conclusions: These findings support the acceptability and preliminary validity of DASI-2. Its inclusive structured observational record may provide a practical complement to resource-intensive tools such as the ADOS-2; however, further validation in larger and more diverse samples is needed.

## 1. Introduction

Our understanding of the diagnosis of autism spectrum disorder (ASD) has evolved considerably over the past decade, driven by a paradigm shift in the understanding of ASD and the increasing demand for diagnostic testing [1,2]. The assessment of ASD in children and young people presents significant challenges for clinicians, families, and healthcare systems [3]. While autism is associated with diverse cognitive and social profiles, current diagnostic systems often frame these differences as impairments.

‘Gold-standard’ diagnostic approaches typically involve multidisciplinary evaluation using tools such as the Autism Diagnostic Observation Schedule, Second Edition (ADOS-2) [4], and the Autism Diagnostic Interview—Revised (ADI-R) [5], alongside observation, detailed developmental histories, and collateral information. These methods are resource-intensive and time-consuming, and access to them is often limited to specialist settings [6]. As demand for ASD assessments grow globally [7], there is a pressing need for diagnostic tools that are clinically robust, accessible, and scalable for wider implementation in community and outpatient services.

Many existing tools are based on algorithmic scoring systems developed through statistical correlations with existing diagnostic groups. Examples include the Autism Diagnostic Interview—Revised (ADI-R) and the Diagnostic Interview for Social and Communication Disorders (DISCO) [8], which apply structured scoring algorithms to generate diagnostic conclusions. While these tools can be valuable in supporting clinical decision making, the alignment to formal diagnostic frameworks is not transparent. Moreover, access to such tools is often restricted by licensing fees, training requirements, and limited language availability, further complicating equitable service delivery.

The Diagnostic Autism Spectrum Interview (DASI-2) [9] was developed to address these limitations. It is a semi-structured clinical interview developed to support healthcare practitioners in the assessment and diagnosis of ASD in individuals >2 years, consistent with both DSM-5 [1] and ICD-11 [10] diagnostic frameworks. The tool guides clinicians through systematic questioning and prompts for detailed behavioral examples to assess core domains of social communication and restricted/repetitive behaviors. The development of DASI-2 involved international collaboration and iterative consultation with professionals working in specialist autism services, many of whom drew on the experiences of autistic individuals and families they supported. It is currently available in six languages, including English, French, Norwegian, Portuguese, Swedish, and Turkish. However, the present study is the first to formally evaluate its acceptability and diagnostic validity.

DASI-2 is freely available; it maps directly onto diagnostic criteria and incorporates developmental trajectories, functional impacts, and contextually informed judgments about masking, compensatory strategies, and environmental accommodations. The interview includes prompts to consider the presence of possible or known comorbid conditions. In addition to the semi-structured symptom interview, DASI-2 includes a brief observational record, offering an ‘in-house’ assessment tool that enables clinicians to capture real-time behavioral information relevant to the diagnostic process.

This study represents the first published evaluation of DASI-2 and its adaptation for use in Hungarian clinical settings. Following a multistep translation procedure into Hungarian, the aims of this study were threefold:

(1) To assess whether DASI-2 is acceptable to implement in practice;
(2) To evaluate the alignment between outcomes of DASI-2 vs. clinical ASD diagnoses applying standard multidisciplinary methods;
(3) To explore the consistency between DASI-2 observational ratings and ADOS-2 classifications.

## 2. Materials and Methods

### 2.1. Participants

Six participants were recruited from an ASD diagnostic center and one participant was recruited from a private practice, both based in Budapest; five had previously received a diagnosis of ASD and two had not. Inclusion criteria were as follows: (1) aged between 6 and 18 years (Hungarian child psychiatric services include 18-year-olds), and (2) having previously received a full clinical assessment for ASD, including an ADOS-2, by a multidisciplinary team including a child psychiatrist. Participants were all monolingual Hungarians. Children with a diagnosis of intellectual disability were excluded. Participants and their families did not receive any financial or other compensation for taking part. See Table 1 for demographic details.

### 2.2. Measures

#### 2.2.1. Diagnostic Autism Spectrum Interview—Version 2 (DASI-2)

DASI-2 guides the assessor to evaluate each core symptom in terms of its developmental onset, pervasiveness across settings, and functional impact, all of which are required by both classification systems. It provides structured prompts for exploring the presence, frequency, and trajectory of symptoms, along with guidance on evaluating compensatory strategies (e.g., masking, scripting), self-initiated adaptations, and environmental accommodations (e.g., family or workplace support) that may reduce the visibility of symptoms.

While both DSM-5 and ICD-11 allow for the influence of contextual factors in clinical formulation, ICD-11 gives more explicit guidance on the role of camouflaging and environmental adjustments in masking functional impairments. The DASI-2 is specifically designed to account for these influences, offering a structured approach to identify cases where ASD-related difficulties may be present but partially hidden due to internal coping mechanisms or external scaffolding. This makes DASI-2 a robust and flexible tool suitable for use under both diagnostic frameworks.

For the purposes of this study, DSM-5-TR criteria were applied via a structured algorithm where the assessor records whether the required number of symptoms have been endorsed (Yes) or not endorsed (No). Endorsement (Yes) is achieved if at least 1 item per category has been rated as ‘present’ or ‘partially present with impairment and/or managed by significant compensation’. Detailed information about the scoring criteria and associated algorithm is described in DASI-2 which can be downloaded free of charge online [9].

For Criterion 1 (Social Communication and Social Interaction), the assessor records (a) if all three categories of Criterion 1 have been endorsed, (b) if they present across multiple settings and contexts, and (c) if they are not better accounted for by general developmental delays.

For Criterion 2 (Restrictive, Repetitive Patterns), the assessor records (a) if at least two out of the four categories of Criterion 2 have been endorsed, (b) if they are present across multiple settings and contexts and (c) if they are not better accounted for by general developmental delays.

In addition, the assessor is then guided to consider whether symptoms have been present since early childhood and whether symptoms cause clinically significant impairment in social, occupational, or other important areas of functioning.

The assessor is then invited to consider the severity of the client’s presentation and need for support. This decision is based on the evidence gathered during the interview and the clinical judgment of the assessor. One of three categories is rated (requiring mild to moderate support, requiring substantial support, requiring very substantial support). DSM-5 provides guidance on making this decision.

For the purposes of the present study, DASI-2 outcomes were recorded dichotomously (diagnosis: yes/no) for comparison with prior clinical diagnostic decisions. The level of support required was also noted.

#### 2.2.2. DASI-2 Observational Record (OR)

In addition to the structured interview, DASI-2 includes an inclusive brief observational record (OR) designed to capture qualitative behavioral information across five domains: (OR1) language and communication profile, (OR2) emotional insight, (OR3) social understanding, (OR4) imagination and creativity, and (OR5) general presentation. The first four domains are rated using a structured qualitative observation system embedded in the interview protocol. Each observation is written up by the assessor based on spontaneous behaviors and responses during the assessment tasks and interaction. The fifth domain has no rated categories and is a space to qualitatively record general and/or other observations considered of relevance.

For OR1–OR4, DASI-2 provides specific descriptors to guide the assessor in classifying observed behavior into one of four qualitative categories. These categories are consistently defined across all domains and follow the same structured approach, ensuring continuity in how difficulty levels are interpreted and rated.

In this study, the four OR categories were reclassified into a numerical ordinal scale for the purpose of exploratory analysis. Scores were assigned as follows: 0 = no difficulty, 1 = some/limited difficulty, 2 = difficulty, and 3 = marked difficulty or inability/refusal to attempt task. This reclassification enabled preliminary evaluation of whether the DASI observational record could be analyzed quantitatively to complement its primary function as a qualitative tool. For example, in OR1 (language and communication profile), assessors rated five subdomains (relating to general language level, conversation flow, direction of conversation, unusual use of language, and non-verbal communication) using the 0–3 descriptors. Each subdomain rating was aggregated to generate an overall score for that domain which was then divided by the number of its subdomain items to generate a mean score for that domain. Applying this process provided a structured ordinal metric of observed functional difficulty to compare within- and between-group variation, including that from other observational tools such as the ADOS-2. The possible minimum and maximum scores for the OR categories are as follows:

1. OR1 (language and communication): Range 0–15;
2. OR2 (emotional insight): Range 0–24;
3. OR3 (social understanding): Range 0–9;
4. OR4 (imagination and creativity): Range 0–1;
5. Total OR Score: Range 0–51.

#### 2.2.3. Adaptation and Scoring Revision

The Hungarian version of DASI was developed by the Research Group of Youth Mental Health, at Eötvös Loránd University, Budapest. A high-quality Hungarian translation was ensured by a formal multistep adaptation process in accordance with the World Health Organization guidelines for translation and adaptation of instruments [11]. The process included forward translation, blind back-translation, expert structured harmonization, pilot testing, and finalization. Discrepancies between the original and back-translated English texts were reviewed by two independent analysts, the translator, and the re-translator. A consensus version was agreed through a point-by-point reconciliation process.

Following this, pilot interviews were conducted with ten mothers; seven with children previously diagnosed with ASD and three with children diagnosed with ADHD. These interviews focused on the clarity and accessibility of interview questions. Their involvement was limited to this adaptation and harmonization stage; it did not extend to the validation described in the procedure section. Parents were contacted through private practices in Budapest, and they received detailed verbal and written information about the purpose of the tool development. They participated in the interview testing voluntarily and anonymously. They did not answer interview questions themselves, nor were their children assessed. They provided feedback on how understandable the items were and whether the language was suitably non-technical. This led to small further refinements of DASI-2 based on this feedback. While no formal user advocacy group was involved, this step ensured meaningful input from individuals with lived experience of autism. Their contributions enhanced the linguistic and contextual relevance of the translated tool.

During early use of the translated tool, complications emerged regarding the application of the original DASI scoring categories, particularly the distinction between “partially present with impairment” and “partially present without impairment.” A refinement was therefore introduced to combine these two categories into a single rating: “partially present with impairment and/or managed by significant compensation.” This change allowed assessors to account for symptoms that were masked or contextually supported and brought the tool into stronger alignment with ICD-11′s recognition of environmental and compensatory influences. This scoring refinement was made for pragmatic and clinical utility reasons, with the aim of improving clarity and reducing ambiguity during real-world administration. The revised version, referred to as DASI-2, was used in the pilot validation. Formal evaluation of the reliability and validity of this revised scoring approach is planned for future studies.

### 2.3. Procedure

For the validation process, researchers initially approached clinicians of patients who had received an ASD assessment in the past and informed them about this study. They were informed that the aim of the study was to conduct a pilot validation of a new clinical interview in Hungary, which involved re-assessment of patient participants by trained researchers using the DASI clinical interview. The clinicians discussed the research with their patients and their parents/caregivers, and those who were open to participate consented for the researchers to contact them directly to discuss this in detail.

Potential participants, both children and their parents/caregivers, were initially contacted by researchers by email. They met with the researchers to discuss their potential participation and ask questions. They were given an information sheet describing the study and invited to take part. Participants and their parents/caregivers were assured that they could withdraw from the study at any time, that all data would be anonymized, and that the outcome of the DASI data would not be disclosed on their medical records. It was emphasized that participation would not impact on the outcome of their previous assessment or influence their ASD treatment in any way. Written informed consent was obtained from parents/caregivers and adolescents >14 years who agreed to participate in the DASI interview and for the researchers to access their clinical records to obtain the outcome of their previous clinical assessment. Participation was voluntary and no incentive or payment was made. Prior to the interview, during the in-person meeting, the children were individually informed again (face-to-face) that they could withdraw from the interview at any time. This was an attempt to counterbalance the potential power dynamics between parent and child.

Consenting participants were assessed using the DASI clinical interview at the Autism Diagnostic Centre in Budapest between March and July 2024. The DASI assessment was administered over two sessions; the first session was an interview with one of the parents (six mothers and one father), while the second session was an interview with the child or young person and the administration of the OR tasks. Following the DASI-2 interview, the researchers accessed the clinical records of each participant to ascertain the outcome of their previous clinical assessment.

Participants were interviewed by either a board-certified child psychiatrist or psychologist, both of whom were PhD students, fully trained in conducting clinical ASD assessments, and in the administration of DASI. The interviewers received continuous supervision from a senior board-certified child psychiatrist member of the study staff. The interviewers were not blinded to the children’s original diagnoses; however, details of their previous clinical assessments were not available to them prior to the interviews. The scoring was always conducted by the same interviewer who had administered DASI-2. If any uncertainties arose, these were discussed with the supervisor.

### 2.4. Statistical Analysis

Data were analyzed descriptively to explore patterns in the observational ratings and their relationship to clinical outcomes. Mean scores across the four observational record domains (OR1–OR4) were calculated for each participant and then aggregated by diagnostic group (ASD vs. non-ASD). Cohen’s kappa was used to estimate agreement between DASI-2 diagnostic outcomes and prior clinical diagnoses.

Given the very small sample size, particularly in the non-ASD group, these analyses are exploratory only and are presented for illustrative and hypothesis-generating purposes. The findings are best viewed as preliminary and indicative rather than conclusive.

## 3. Results

### 3.1. Implementation Acceptability

The DASI-2 interview was successfully completed by all participants in the pilot sample. Researchers were able to administer the full interview protocol, apply the scoring system (including the revised DASI-2 categories), and complete the observational records without missing data. Following training and adaptation, the tool was reported to be usable in its current format, and post-interview discussions among the research team allowed for reflection on both its strengths and limitations in practice. These findings suggest that the tool is acceptable and practical for use in clinical and research settings and may be well suited for integration into multidisciplinary assessment frameworks.

#### Pilot Validation of DASI-2

Table 2 presents the DASI-2 outcomes compared with prior multidisciplinary clinical diagnoses. In six out of seven cases, the DASI-2 outcome was aligned with the previous clinical diagnosis (i.e., five participants were diagnosed with ASD and one participant was not diagnosed with ASD). There was disagreement regarding the diagnosis of Participant 3. To quantify agreement, Cohen’s kappa coefficient was calculated, yielding a value of κ = 0.70, indicating substantial agreement between DASI-2 and clinical diagnostic conclusions. Given the small sample size, this result should be interpreted with caution, but it provides preliminary support for the diagnostic consistency of the tool.

In the one case where outcomes did not align (Participant 3), the DASI-2 interview did not confirm a diagnosis of ASD, although the previous clinical team had done so. The reason was because DASI-2 had coded two key symptoms as absent (integration of verbal and non-verbal communication and use of spontaneous non-verbal communication). To examine this further, assessment notes of the previous clinical assessment and DASI-2 were compared. This revealed no discrepancy as the previous clinical assessment had also noted that these symptoms were absent. This meant that both the previous assessment and DASI-2 should have reached diagnostic agreement. It was also noted that the ADOS-2 outcome administered as part of the previous clinical assessment was also not indicative of ASD. It seems that the divergence in diagnostic outcome was attributed to the previous clinical assessment overriding the interview outcome (of ASD not being present) due to the additional contextual information available to the clinical team, teacher reports, and school-based observations, which were not available for the DASI-2 interview. This suggests that the difference arose not from disagreement in symptom-level interpretation at interview stage, but from differences in the scope of information available to each assessor which influenced the final diagnostic decision. This case illustrates that structured tools such as DASI-2 play an important role in supporting the diagnostic process, but they do not replace the need for comprehensive clinical judgment and contextual understanding.

### 3.2. Comparison of Observation Ratings by Diagnostic Outcome

The DASI observational ratings are designed to provide a structured method of observation for the assessor. However, to explore their utility by applying dimensional ratings, the mean scores generated from the structured observational record (OR1–OR4) were compared between individuals who did and did not receive a clinical diagnosis. As shown in Table 3, participants diagnosed with ASD (n = 5) consistently obtained higher scores across all four observational domains. The largest differences were seen in OR1 (language and communication profile) and OR3 (social understanding), where mean values were substantially higher in the ASD group. The total observational scores (calculated as the sum of individual means across OR1 to OR4) were also substantially higher for the ASD group (total mean = 15.26) compared to the non-ASD group (total mean = 1.57).

Further comparison was made with the ADOS-2 classifications derived from the participants’ initial clinical assessments. Among the five participants whose ADOS classifications were consistent with a diagnosis of ASD, four were rated as having moderate symptom severity, while Participant 5 received a classification of high severity, indicating a requirement for substantial support. Notably, this individual also recorded the highest total observational score on the DASI (mean = 4.67 across OR1–OR4), further supporting alignment between these tools.

In contrast, the two participants whose ADOS classifications did not support an ASD diagnosis also had the lowest DASI observational scores (Participant 3: mean = 1.37; Participant 4: mean = 0.20). This correspondence between DASI observational data and ADOS classifications provides preliminary evidence of convergent validity between the two tools.

## 4. Discussion

This study presents the adaptation of DASI-2 into Hungarian and its small pilot validation in a clinical setting. The Hungarian adaptation of DASI-2 was successfully completed, including a multistep process, cultural and contextual adaptation, and preliminary field testing in clinical settings to assess clarity, relevance, and acceptability. Feedback from clinicians and parents was incorporated to fine-tune the language and structure of the items, ensuring both its integrity with the original tool while making it accessible for Hungarian clinicians and patients alike.

All three aims of this study were met: DASI-2 was found to be acceptable to administer in clinical practice; its diagnostic outcomes showed alignment with prior multidisciplinary assessments; and its observational ratings corresponded closely with ADOS-2 classifications. The sample is small, but findings offer promising early support for the tool’s clinical utility and diagnostic alignment, as well as for the potential value of its structured observational component.

DASI-2 was implemented acceptably in practice. The interview was completed in full with all participants, and researchers were able to apply the scoring system and complete observational records without missing data. Post-assessment reflections also highlighted the tool’s usability in clinical and research settings.

The pilot validation demonstrated substantial agreement between DASI-2 diagnostic outcomes and prior multidisciplinary clinical diagnoses, with agreement in 6 of the 7 cases and a Cohen’s kappa coefficient of 0.70. In the one case where outcomes differed, both the DASI-2 and the clinical assessment had rated key non-verbal communication symptoms as absent. The difference in diagnostic decision appeared to result from additional contextual information (e.g., teacher reports, school observations) available to the clinical team, but not to the DASI-2 assessor. Of note, the ADOS-2 outcome for this participant was consistent with the DASI-2 outcome. This finding highlights the importance of interpreting structured interview data within a broader, multi-method diagnostic process [12]. Importantly, it does not undermine the DASI-2′s validity; rather, it affirms that the interview generated consistent symptom-level ratings when compared with clinical opinion.

In addition to validation, this study explored the use of the DASI-2 observational record (OR) as a structured tool for recording qualitative information across four domains: language and communication, emotional insight, social understanding, and creativity. Each OR domain was rated using a consistent four-point qualitative scale and was subsequently recoded into an ordinal scoring system to explore its potential quantitative value. The results showed a clear pattern: participants diagnosed with ASD obtained higher OR scores across all domains, with a total mean score of 15.26 compared to 1.57 in those not diagnosed. The strong alignment with ADOS-2 classifications further supports the construct validity of the OR scores. For example, the individual classified by ADOS-2 as having high-severity ASD also obtained the highest DASI-2 observational score. Conversely, the two participants whose ADOS classifications did not support an ASD diagnosis also obtained the lowest DASI-2 OR scores, providing a meaningful degree of convergence between the tools.

These findings suggest that the DASI-2 observational record may offer a brief, structured supplement to traditional interview-based assessment, with potential to capture clinically relevant behavioral information in a format that is both scalable and compatible with real-world diagnostic practice. While originally designed as a qualitative aid to formulation, the exploratory quantification of OR scores in this study offers a promising direction for future research, particularly for use in contexts where comprehensive observational tools like the ADOS-2 are not available. Nonetheless, as with all observational assessments (structured or unstructured), there are inherent limitations, including the potential for subjective bias and contextual dependency. Factors such as assessor experience, environmental setting, and the nature of the assessment interaction may all influence the behaviors observed and how they are interpreted [13]. These limitations underscore the need for observational data to be considered alongside developmental history, informant reports, and clinical judgment.

### Limitations

This pilot study had several limitations that constrain the generalizability of the findings. The sample size was very small, particularly in the non-ASD comparison group which included only two participants. This precluded statistical inference and restricted interpretation to a purely exploratory level. The observed patterns should therefore be regarded as hypothesis-generating rather than confirmatory, offering preliminary insights to guide future, larger-scale validation efforts. The small sample also limited representation of the diversity and heterogeneity of the autistic population, particularly with respect to gender, non-binary identities, and cultural variation [13,14]. Additionally, while participants were recruited from both public and private settings, detailed demographic information such as ethnicity and socioeconomic background was not systematically recorded, limiting the ability to assess sampling diversity.

Further, the observational ratings were derived through retrospective recoding of qualitative data and have not yet been subjected to formal psychometric validation. While this exploratory quantification represents an innovative step toward structured behavioral assessment, the scores should be interpreted with caution due to potential measurement error and a lack of established construct validity. Similarly, although the observational component showed good agreement with ADOS-2 outcomes, individual item-level alignment was not analyzed, and the tool’s performance in more diverse clinical contexts remains unknown.

This study also did not implement a blinded design. Assessors conducting the DASI-2 interviews were not blind to participants’ previous diagnoses, raising the possibility of confirmation bias. While steps were taken to ensure standardized administration, future studies should consider blinded procedures to strengthen methodological rigor.

Although parent feedback informed the translation and adaptation of the Hungarian version of DASI-2, no formal involvement from autistic individuals or advocacy groups was included during the validation phase. Broader co-production and involvement of neurodivergent voices in all stages of development would enhance the cultural validity and relevance of the tool.

Finally, it is important to acknowledge the limitations inherent to structured diagnostic tools themselves. While DASI-2 aims to support diagnostic decision making, structured interviews cannot fully capture the breadth of lived experience or account for individual variability in presentation due to masking, context, or cultural factors. As such, tools like DASI-2 should be viewed as one part of a broader, person-centered diagnostic process, embedded within a neurodiversity-affirming framework.

## 5. Conclusions

In summary, this study supports the acceptability and preliminary validity of DASI-2 as a clinical interview for the assessment of ASD. DASI-2 was successfully adapted for use in Hungarian-speaking populations, and the outcomes of this pilot study support its utility in future psychometric evaluation with larger samples. In time, the Hungarian version of DASI-2 may offer healthcare professionals in Hungary a structured, diagnostically aligned tool for the assessment and formulation of ASD. Future research should include participatory approaches involving autistic individuals in the evaluation and refinement of DASI-2.

This study also introduces early evidence that DASI-2′s observational record may offer an efficient and meaningful way to document behavioral features during assessment. The tool demonstrated alignment with both clinical diagnoses and ADOS-2 outcomes in this small pilot sample.

In addition to its structured design and conceptual alignment with diagnostic criteria, DASI-2 offers practical advantages: it is freely available, making it cost-efficient and accessible to a wide range of services, including those with limited resources. Unlike some other measures that rely on statistical associations with ASD, DASI-2 is explicitly mapped to diagnostic criteria, supporting clear, criterion-based formulation and clinical decision making. These features, together with its ease of use and promising early performance, justify further validation in larger, diverse populations and suggest that DASI-2 may serve as an alternative or complement to more resource-intensive tools within multidisciplinary diagnostic pathways.

## Figures and Tables

**Table 1:** Demographic and treatment details of participants for the validation process of DASI.

	Age at the Time of ADOS Assessment (Years)	Age at the Time of DASI Assessment (Years)	Sex	Father′s Educational Level	Social-Communication Skills Training Received Before or During the ADOS Assessment?	Social-Communication Skills Training Received During the DASI-2 Assessment?
Participant 1	15.5	16.2	female	no data available	no	no
Participant 2	8.10	13.6	male	university degree	yes	no
Participant 3	16.4	16.11	female	university degree	no	no
Participant 4	15.2	15.8	male	university degree	no	no
Participant 5	16.0	17.9	male	university degree	no	no
Participant 6	9.3	10.0	male	college degree	yes	yes
Participant 7	16.6	16.8	female	high-school graduation	no	no

**Table 2:** DASI-2 outcomes compared with prior multidisciplinary clinical diagnoses.

	Outcome of Clinical Diagnosis	Content of Clinical Diagnosis	ADOS-2 Classification and Level of Autism Spectrum-Related Symptoms	Outcome of DASI-2 Diagnosis and Level of Support Required
Participant 1	ASD	Clinical diagnostic interview with parents Clinical diagnostic interview with child ADOS-2 3. module School report School behavior observation	Module 3: Autism (moderate)	ASD (mild/moderate)
Participant 2	ASD	Clinical diagnostic interview with parents Clinical diagnostic interview with child ADOS-2 3. module School report School behavior observation	Module 3: Autism spectrum (moderate)	ASD (mild/moderate)
Participant 3	ASD	Clinical diagnostic interview with parents Clinical diagnostic interview with child ADOS-2 4. module School report School behavior observation	Module 4: Non-spectrum	NO ASD
Participant 4	NO ASD	Clinical diagnostic interview with parents Clinical diagnostic interview with child ADOS-2 4. module School report School behavior observation	Module 4: Non-spectrum	NO ASD
Participant 5	ASD	Clinical diagnostic interview with parents Clinical diagnostic interview with child ADOS-2 3. module School report School behavior observation	Module 3: Autism (high)	ASD (substantial)
Participant 6	ASD	Clinical diagnostic interview with parents Clinical diagnostic interview with child ADOS-2 3. module School report School behavior observation	Module 3: Autism spectrum (moderate)	ASD (mild/moderate)
Participant 7	ASD	Clinical diagnostic interview with parents Clinical diagnostic interview with child ADOS-1 4. module	Module 4: Autism spectrum (moderate)	ASD (mild/moderate)

**Table 3:** Comparison of observation ratings for individuals with and without a DASI-2 ASD diagnosis.

	OR1 Mean	OR2 Mean	OR3 Mean	OR4 Mean	TOTAL Mean	ADOS-2 Outcome from Previous Clinical Assessment
Participant 1	1.20	0.75	0.33	0.25	2.53	Module 3 Classification: autism Level: moderate Support Required: mild/moderate
Participant 2	1.20	0.75	0.67	0.25	2.87	Module 3 Classification: autism spectrum Level: moderate Support Required: mild/moderate
Participant 5	2.75	0.25	1.67	0.00	4.67	Module 3 Classification: autism Level: high Support Required: substantial
Participant 6	1.00	0.25	0.67	0.25	2.17	Module 3 Classification: autism spectrum Level: moderate Support Required: mild/moderate
Participant 7	0.80	0.62	1.60	0.00	3.02	Module 4 Classification: autism spectrum Level: moderate Support Required: mild/moderate
ASD total	6.95	2.62	4.94	0.75	15.26	
Participant 3	0.20	0.50	0.67	0.00	1.37	Module 4 Classification: non-spectrum
Participant 4	0.20	0.00	0.00	0.00	0.20	Module 4 Classification: non-spectrum
Not ASD total	0.40	0.50	0.67	0.00	1.57	

## Data Availability

The original contributions presented in this study are included in the article. For reasons related to the preservation of patient privacy, further inquiries can be directed at the corresponding author.
